# An Efficient Seam Elimination Method for UAV Images Based on Wallis Dodging and Gaussian Distance Weight Enhancement

**DOI:** 10.3390/s16050662

**Published:** 2016-05-10

**Authors:** Jinyan Tian, Xiaojuan Li, Fuzhou Duan, Junqian Wang, Yang Ou

**Affiliations:** 1 Beijing Key Laboratory for Geographic Information Systems and Environment and Resources, Capital Normal University, Beijing 100048, China; 2130901016@cnu.edu.cn (J.T.); lixiaojuan@cnu.edu.cn (X.L.); 2150902042@cnu.edu.cn (Y.O.); 2 Department of Geography and Environmental Management, University of Waterloo, Waterloo, ON N2L 2R7, Canada; j563wang@uwaterloo.ca

**Keywords:** UAV, Wallis dodging, seam elimination, Gaussian distance weight enhancement, earthquake

## Abstract

The rapid development of Unmanned Aerial Vehicle (UAV) remote sensing conforms to the increasing demand for the low-altitude very high resolution (VHR) image data. However, high processing speed of massive UAV data has become an indispensable prerequisite for its applications in various industry sectors. In this paper, we developed an effective and efficient seam elimination approach for UAV images based on Wallis dodging and Gaussian distance weight enhancement (WD-GDWE). The method encompasses two major steps: first, Wallis dodging was introduced to adjust the difference of brightness between the two matched images, and the parameters in the algorithm were derived in this study. Second, a Gaussian distance weight distribution method was proposed to fuse the two matched images in the overlap region based on the theory of the First Law of Geography, which can share the partial dislocation in the seam to the whole overlap region with an effect of smooth transition. This method was validated at a study site located in Hanwang (Sichuan, China) which was a seriously damaged area in the 12 May 2008 enchuan Earthquake. Then, a performance comparison between WD-GDWE and the other five classical seam elimination algorithms in the aspect of efficiency and effectiveness was conducted. Results showed that WD-GDWE is not only efficient, but also has a satisfactory effectiveness. This method is promising in advancing the applications in UAV industry especially in emergency situations.

## 1. Introduction

The development of UAVs conforms to the current increasing demand for low-altitude very high resolution (VHR) remote sensing data [1,2,3]. Compared with the traditional photogrammetry process, the fast reconstitution of UAV image mosaics is a precondition of its application [4,5]. However, the UAV image-processing challenges include large geometric deformity, small size, large number and uneven exposure. These challenges lead to difficulties in seam elimination when mosaicking UAV images [6,7]. The mosaic seams mainly come from two sources: (1) the color or brightness differences due to the exposure variation; and (2) the texture misplacement due to geometric deformity, projection differences caused by tall landscapes and image capture position differences [8]. These two types of seams clearly appear on the UAV remote sensing platform, therefore, the effective and efficient removal of these seams is essential for the application of UAVs.

At present, the major methods of seam elimination are the seamline detection and image fusion methods. The seamline detection method should be considered as a way of circumventing the problem of tall landscapes in the images [9], and can be attributed to two categories: the first category is seamline search by the variation of gradient degree or image texture. Davis [10] proposed the optimal seamline searching method based on Dijkstra’s algorithm, which relies mainly on the calculation of adjacency matrices and distance matrices of high algorithmic complexity [11]. Yuan [12] replaced the Dijkstra algorithm with a greedy algorithm for local optimal path selection. However, the algorithm was still influenced by iterative convergence. Kerschner [13] applied the twin snake operator to automatically select the image seamline. However, the operator cannot guarantee the systematical optimization. Chon [14] eliminated seamlines by dynamic planning stitching. The computational burden of the algorithm rises exponentially with the increase of seamline length [15]. The second category is applying ancillary data to detect the seamline. Wan [16] proposed an algorithm based on the vector path ancillary data, which is only suitable for a few systems and is significantly limited by the vector data. Zuo [17] applied the greedy snake algorithm with the assistance of the DSM method to detect seamlines. The algorithm is fairly complicated and highly dependent on the ancillary data. In conclusion, all these searching seamline algorithms applied on UAV images have three limitations: (1) they require high geometric accuracy of the UAV images, but UAV remote sensing platforms are rather instable and have low parameter accuracy. The equipped camera sensors cannot meet the accuracy requirements because they are not designed for photogrammetry; (2) All of them are complicated and time-consuming. UAV images are small in size but contain large amounts of data, which requires high processing efficiency; (3) Objects in UAV images are not overlapped in a regular manner. The seamlines are difficult to detect, especially for regions with high densities of tall buildings.

In addition to the seamline detection method, image fusion can also be applied to eliminate mosaic seams [18]. Uyttendael [19] applied a feathering and interpolating function based on weighted features to reduce the color difference. However, the feathering algorithm tends to give fuzzy edges when smoothing the exposure difference, and can sometimes lead to the “ghosting” effect. Szeliski [20,21] manually selected at least four pairs of feature points, and estimated the variation of images with the function built on the variation of pixel difference of the feature points, which achieved a satisfactory layer fusion effect. However, since the estimation is based on brightness differences, it is highly sensitive to the brightness of images and can be poorly automated [22]. Su [23] proposed an image fusion method based on wavelet multi-scale decomposition. This method first applies wavelet multi-scale decomposition over the source images. Then, the wavelet weight parameters are determined and the images are reconstructed through inverse wavelet transform. The algorithm is highly complicated and it is difficult to determine wavelet parameters [24]. Zomet [25] eliminated mosaic seams by analyzing the contrast in smooth stitching areas. However, the field smoothing can lead to the appearance of “ghosting” effects [11,26]. Tian [27] developed a brightness and texture seam elimination (BTSE) method with a smooth transition effect on a one-dimensional direction in the overlap region. A “ghosting” effect tends to appear at the border when the algorithm is applied to UAV images with the large geometric deformity. In conclusion, all these image fusion methods for UAV images have two major limitations: (1) a “ghosting” effect tends to appear due to the uneven exposure and the large geometric deformity of UAV images; (2) they are fairly complicated and require long computation times, which conflicts with the fact that UAV systems require high data processing efficiency to deal with the massive amount of image data.

Therefore, the objective of this study is twofold: firstly, to adjust the difference of brightness between the two matched images with the Wallis dodging method and; secondly, to develop a new image fusion algorithm to eliminate the texture seamline based on the First Law of Geography.

## 2. Study Site and Data

The study site is located in Hanwang (104°09′E to 104°12′E and 31°25′N to 31°28′N) in the northwestern part of the Sichuan Basin (China) and has an overall area of 54.3 km^2^. It is a city at the foot of mountains with an average elevation of 685 m above sea level and slopes of less than 5°. As an industrial city, it has a sound transportation system and a total population of 53,000, among which the non-agricultural population is 35,000 [28,29]. The major land uses of this study site are woodland, farmland, water, road, and buildings. In this task, UAV image data were acquired on 15 May 2008 after the 5.12 Wenchuan Earthquake. The flight altitude and speed of the UAV platform are 400 m and 50 km/h, respectively. The major parameters of the image sensor equipped on the UAV platform are shown in Table 1. A total of 678 images were acquired with an image resolution of 0.3 m. The average forward overlap is 70% and the side forward overlap is 40%.

## 3. Methodology

### 3.1. Wallis Dodging

Image processing before image fusion contains two major steps: image matching and image dodging. Image matching aims to find corresponding points, and image dodging was used to eliminate the brightness differences between two matched images. First, in order for us to find the corresponding points between two images, an image matching method should be applied. In this study, the Scale-Invariant Feature Transform (SIFT) algorithm was used to match the two images [30,31], which consists of four stages: (1) building the scale-space; (2) keypoint localization; (3) removal of bad keypoints; and (4) keypoint description. It has been proven in many studies [32,33] that SIFT not only performs well in image rotation, scale zoom and illumination changes, but also does well in affine transformation, and noise jamming. Subsequently, the Random Sample Consensus (RANSAC) method was applied to the points matched by SIFT to remove any mismatched points [34]. Additionally, the Wallis dodging algorithm [8,35,36] was employed to adjust the difference of brightness between the two matched images before the texture seam elimination method.

The principle behind Wallis image dodging is that it can adjust the variance and mean value of the target image to the reference image’s level. The Wallis filter can be defined by Equation (1):

(1) $$I_{ij}=(I_{ij}^{2}-\bar{I^{2}})\times \frac{c\sigma_{I^{1}}}{c\sigma_{I^{2}}+(1-c)\sigma_{I^{1}}}+b\bar{I^{1}}+(1-b)\bar{I^{2}}$$

where *I*^1^ is reference image, *I*^2^ is target image, and *I_ij_* is the pixel value of *I*^2^ in *i* row, *j* column after image dodging. $\bar{I^{1}}$, $\bar{I^{2}}$ and $\sigma_{I^{1}}$, $\sigma_{I^{2}}$, are the mean and variance value of *I*^1^ and *I*^2^, respectively; *c*∈[0.1] is an adjustment coefficient for variance value of the image, and *b*∈[0.1] is an adjustment coefficient for the mean value. However, setting the two specific parameters is still a critical question in the existing research. The parameter setting method was derived in this study. First, the variance of the target image is shown in Equation (2):

(2) $$\sigma_{I^{2}}=\sqrt{\sum_{i=0}^{m-1}\sum_{j=0}^{n-1}(I_{ij}^{2}-\bar{I^{2}}{)}^{2}/m\times n}$$

Second, the variance and mean value of the target image was adjusted to the reference image’s level. So the variance and mean value of the target image after image dodging should be roughly equal to $\sigma_{I^{1}}$ and $\bar{I^{1}}$, respectively. Therefore, they can be denoted as Equation (3):

(3) $$\sigma_{I^{1}}\approx \sqrt{\sum_{i=0}^{m-1}\sum_{j=0}^{n-1}(I_{ij}-\bar{I^{1}}{)}^{2}/m\times n}$$

Third, both sides of the Equation (3) are multiplied by $\sigma_{I^{2}}/\sigma_{I^{1}}$:

(4) $$\sigma_{I^{2}}\approx \frac{\sigma_{I^{2}}}{\sigma_{I^{1}}}\times \sqrt{\sum_{i=0}^{m-1}\sum_{j=0}^{n-1}(I_{ij}-\bar{I^{1}}{)}^{2}/m\times n}$$

Then, simultaneous application of Equations (2) and (4) gives:

(5) $$\frac{\sigma_{I^{2}}}{\sigma_{I^{1}}}\times (I_{ij}-\bar{I^{1}})\approx I_{ij}^{2}-\bar{I^{2}}$$

Finally, the pixel value of target image after image dodging is shown in Equation (6):

(6) $$I_{ij}\approx \frac{\sigma_{I^{1}}}{\sigma_{I^{2}}}\times (I_{ij}^{2}-\bar{I^{2}})+\bar{I^{1}}$$

Comparing Equation (1) with Equation (6), it found that we will get Equation (6) when the parameters (*b* and *c*) were both set to 1 in Equation (1). Therefore, to adjust the mean and variance value of target image to reference image’s level, Equation (6) with Wallis filter (*b* = 1, *c* = 1) was used for UAV image dodging.

### 3.2. GDWE Method

#### 3.2.1. Theoretical Basis

The First Law of Geography proposed by Waldo Tobler in 1970 is “all attribute values on a geographic surface are related to each other, but closer values are more strongly related than are more distant ones” [37]. The law is the foundation of the fundamental concepts of spatial autocorrelation and spatial dependence [38], based on which we have developed an effective and efficient seamline elimination method (GDWE) for UAV image. The principle of GDWE is an image fusion algorithm combining relevant information from two matched UAV images into a single image in the overlapping region. As such, GDWE embraces three major steps: first, the principal point of each image was set as the optimal pixel with the minimum geometric distortion because the image sensor equipped on UAV platform is, in general, a type of non-measurement array CCD camera. Second, the weight in a certain pixel contributed by each image in the overlap region was determined by the distance between the pixel and the principal point. A two-dimensional Gaussian kernel was then employed to describe it. Third, in order to enhance the influence of distance to the weight, an exponent form adjustment coefficient was introduced and it was parameterized by a sensitive analysis method.

#### 3.2.2. Seam Elimination

**Figure 1:**
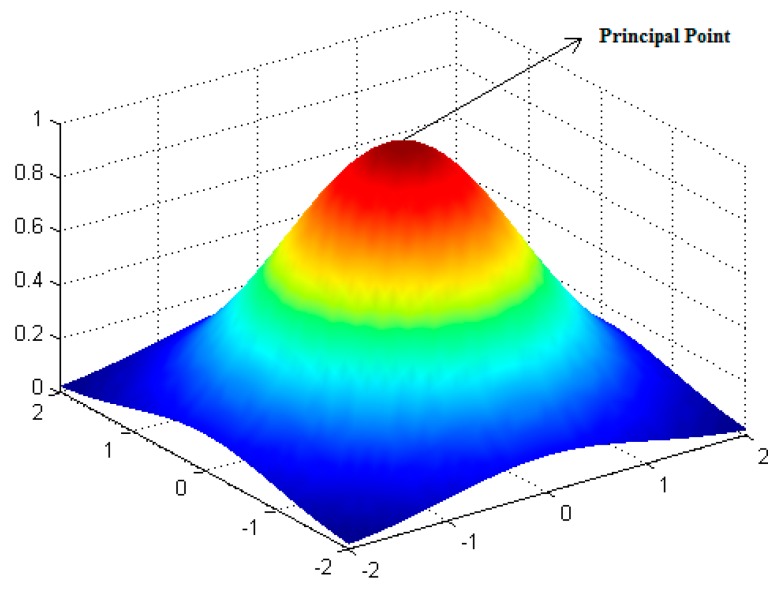
An example of a two-dimensional Gaussians distance weight distribution kernel.

To develop the algorithm for image fusion in the overlap region of the matched UAV images, some parameters should be defined first, in which the principle points of the two matched images were *O*_1_ and *O*_2_; *O* is an arbitrary point in the overlap region; *d*_1_(|*O* − *O*_1_|) and *d*_2_(|*O* − *O*_2_|) are the distances between *O*_1_, *O*_2_ and *O*; The pixel values of point *O* in the two matched UAV images are $I_{ij}^{1}$ and $I_{ij}^{2}$. The pixel value of point *O* after image fusion is *I_ij_*. Therefore, *I_ij_* can be defined as $\omega_{1}\times I_{ij}^{1}+\omega_{2}\times I_{ij}^{2}$, where *w*_1_, *w*_2_ are the weight contributions of the two UAV images to point *O*, and *w*_1_ plus *w*_2_ is equal to 1. Based on the theory mentioned above, a Gaussian kernel shown in Figure 1 was introduced to describe the Gaussian distance weight distribution (*G*_*w*1_), and was defined by Equation (1):

(7) $$G_{w_{1}}=a\times e^{-{\left(\left|O-O_{1}\right|/\left|O-O_{2}\right|\right)}^{2}/2\sigma^{2}}$$

where *a* was set to 1 because *G*_*w*1_ should be equal to 1 when *d*_1_ is 0. In order to enhance the influence of Gaussian distance on the weight, an exponent form adjustment coefficient (*λ*) was introduced into Equation (2):

(8) $$w_{1}=e^{-{\left(\left|O-O_{1}\right|/\left|O-O_{2}\right|\right)}^{2\lambda }/2\sigma^{2}}$$

In which *w*_1_ was set to 0.5 when *d*_1_ equals *d*_2_. When we apply the relationship to Equation (8), we get:

(9) $$\sigma =\sqrt{1/\left(2\times \text{ln}2\right)}$$

Therefore, including these terms in Equation (8) results in Equation (9), the pixel value was defined by Equation (10):

(10) $$I_{ij}=\left(0.5^{{\left(\left|O-O_{1}\right|/\left|O-O_{2}\right|\right)}^{2\lambda }}\right)\times I_{ij}^{1}+\left(1-0.5^{{\left(\left|O-O_{1}\right|/\left|O-O_{2}\right|\right)}^{2\lambda }}\right)\times I_{ij}^{2}$$

Finally, we named our method Wallis dodging and Gaussian distance weight enhancement (WD-GDWE) when taking the Wallis dodging algorithm into consideration. It is shown in Equation (11):

(11) $$I_{ij}=\left(0.5^{{\left(\left|O-O_{1}\right|/\left|O-O_{2}\right|\right)}^{2\lambda }}\right)\times I_{ij}^{1}+\left(1-0.5^{{\left(\left|O-O_{1}\right|/\left|O-O_{2}\right|\right)}^{2\lambda }}\right)\times \left(\frac{\sigma_{I^{1}}}{\sigma_{I^{2}}}\times (I_{ij}^{2}-\bar{I^{2}})+\bar{I^{1}}\right)$$

## 4. Results and Discussion

### 4.1. Wallis Dodging

To assess the efficiency and effectiveness of WD-GDWE for seamline elimination of UAV images, the method was implemented with Visual C++ programming using 8 GB memory and an Intel Xeon 2.5 GHz CPU. The UAV images covering five different types of land use (woodland, farmland, water, road, and buildings) from the study site were tested.

**Figure 2:**
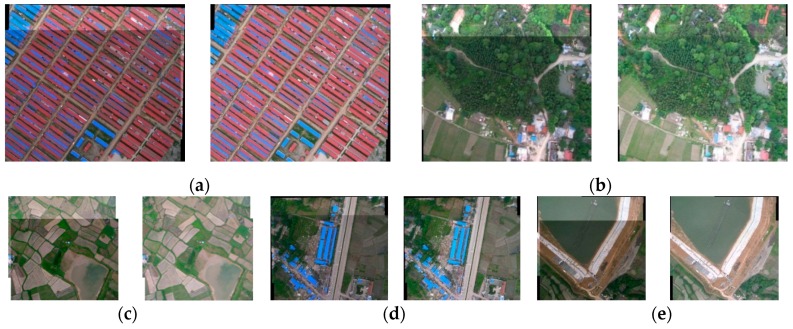
The results of Wallis dodging for two matched UAV images of each type land use, in which (**a**)–(**e**) correspond to buildings, woodland, farmland, road, and water, respectively. For example, in the case of (**a**), the left figure was the direct stacking result of two matched images, whereas the right figure was the stacking result of two matched images after Willis dodging.

Figure 2 shows the results of stacking directly *versus* stacking after Wallis dodging for two matched UAV images covering five different types of land use. From the perspective of visual effects, the results indicate that the brightness difference of two matched images has been effectively balanced by Wallis dodging, in which the left figure of each figure group in Figure 2 was stacked directly and the right figure was stacked after Wallis dodging. The root mean-square error (RMSE) values of the mean and standard deviation were calculated from the two matched UVA images in the overlap region for direct stacking and Wallis dodging, respectively. For each type of land use, at least 36 pairs of matched images were tested, and the averages of the RMSE values were recorded in Table 2. The results show that the Wallis dodging method can effectively balance the brightness differences between the two matched images, in which the RMSE of mean and stand deviation were determined to be 0.0 and less than 0.3, respectively.

### 4.2. WD-GDWE Method

To acquire the optimal adjustment coefficient (*λ*) for the WD-GDWE method, a series of values from zero to five with a step size of 0.2 were set, based on which the optimal value of *λ* was determined when the lowest RMSE between the test images and reference images was achieved. In this study, the optimal value of *λ* was set to 2.6. Lastly, performance comparisons between WD-GDWE and five other classical seamline elimination algorithms were conducted in terms of efficiency and effectiveness. The specific five classical methods are: Tian’s BTSE algorithm, Uyttendael’s feathering algorithm, Su’s Wavelet algorithm, Szeliski’s algorithm, and Davis’s Dijkstra algorithm, in which the first four methods are based on image fusion and the last one is based on seamline detection. Generally, the image quality assessment indicators for seamline elimination can be divided to three types [39,40,41,42,43]: (1) amount of information: information entropy, standard deviation, cross entropy, signal to noise ratio, and joint entropy [44,45]; (2) image quality: average gradient and wavelet energy ratio [46]; (3) spectral information reserved: RMSE, standard deviation, deviation, and spectral distortion; Taking all three types of indicators into consideration, information entropy, average gradient, and RMSE were selected to access the specific five methods of seamline elimination, respectively. In addition, processing time is also an indicator for evaluating the efficiency of the algorithm. It should be noted that orthoimages were severed as reference images of the RMSE, which was produced from the control points recorded by artificial with the help of a differential GPS.

**Figure 3:**
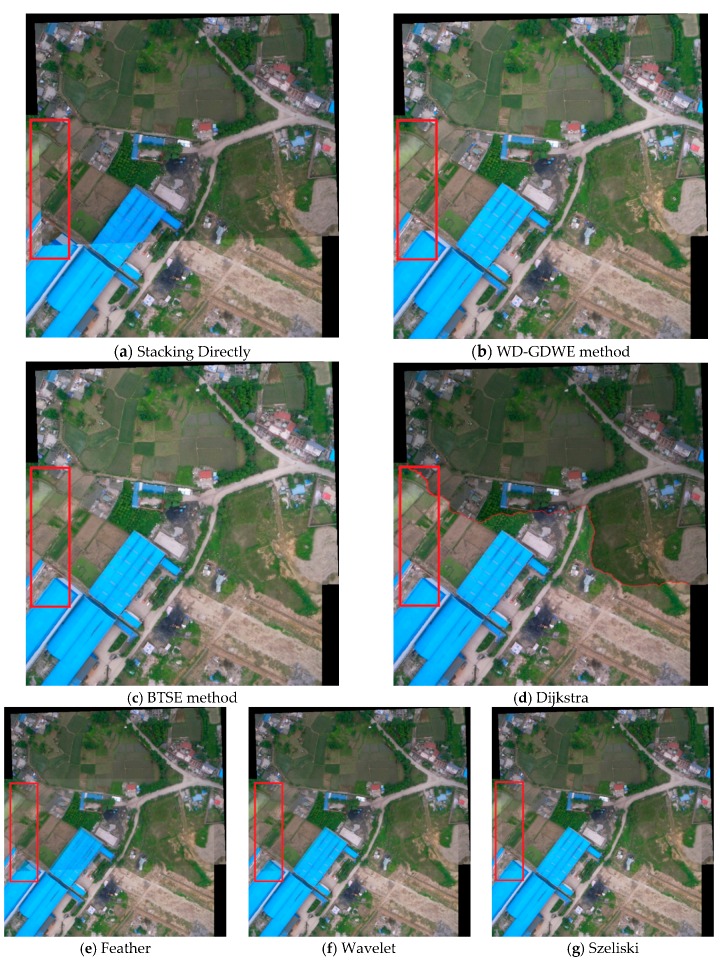
The performance comparison of different seam elimination algorithms.

From the perspective of visual effects, Figure 3 shows the performance comparisons of the five seamline elimination methods, in which Figure 3a is the direct stacking result, Figure 3b is the WD-GDWE method result, Figure 3c–g is the results of the other five different seamless methods, respectively.

**Figure 4:**
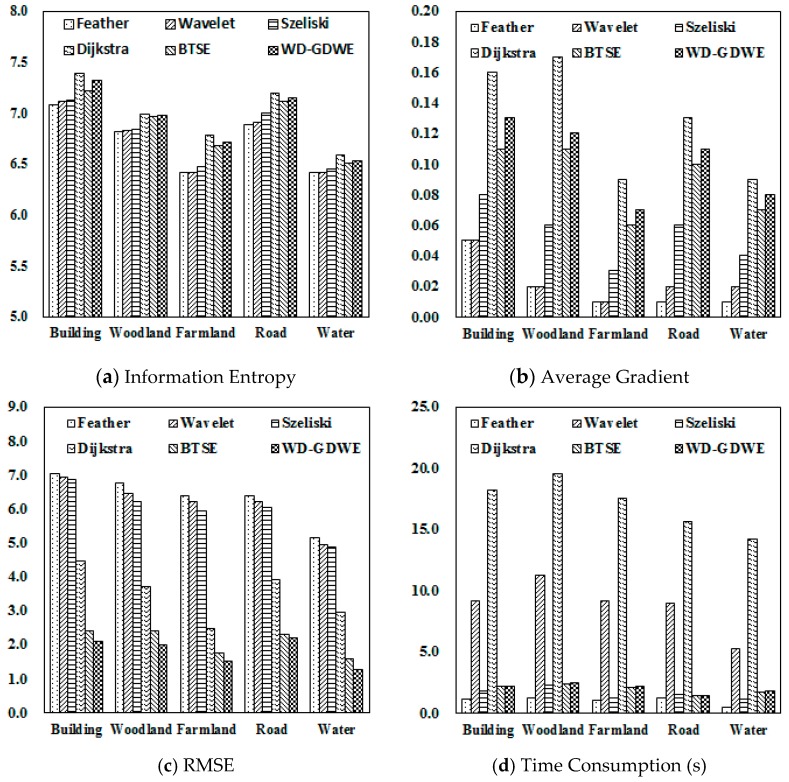
Comparisons of different elimination seam algorithms. (**a**) Information entropy for describing the amount of information; (**b**) verage gradient to access the image qualities; (**c**) RMSE between the specific five methods with the orthoimages; (**d**) time consumption of the six methods.

**Figure 5:**
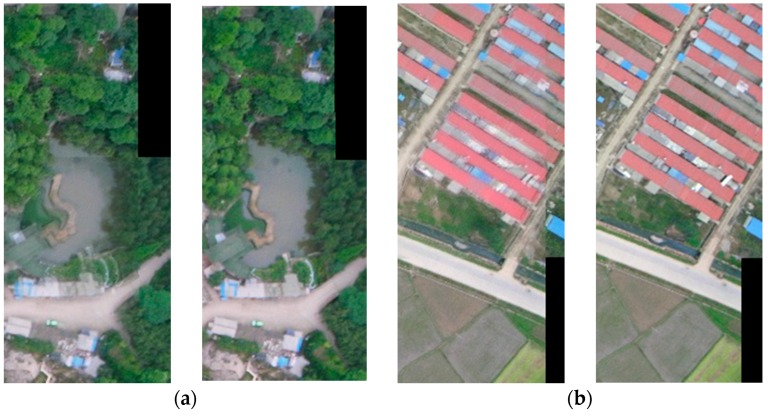
Both (**a**) and (**b**) are the results at the border of the fusion images, in which the left one in (**a**) or (**b**) is with the BTSE method and the right one used the WD-GDWE method.

Comparing Figure 3a,b, we find that the buildings and the roads obviously display mosaic dislocation, whereas the phenomenon has been greatly improved with the WD-GDWE method. The performance comparisons of the five seamline elimination methods shown in Figure 3c–f indicate that: (1) a “ghosting” effect tends to appear in the Feather, Wavelet, Szeliski, and BTSE algorithms; (2) the visual effects of the Dijkstra algorithm and WD-GDWE are much better than those of the other methods. From the perspective of image quality assessment indicators, the details of the performance comparisons of the six methods were shown Figure 4. Each of the four indicators is an average value calculated from lots of UAV images (at least 36 pairs) for each type of land use. Figure 4a,b show that Dijkstra method gives the most abundant amount of information and the highest definition, and the WD-GDWE method follows. The BTSE is worse than the WD-GDWE method at the border of the fusion image because it only supports smooth transitions in a one-dimensional direction in the overlap region. Considering the improvement of WD-GDWE from BTSE is not obvious in Figure 3 from the perspective of visual effects, therefore, some experimental results at the border of the fusion images with the two methods were added (Figure 5). The Wavelet and Szeliski algorithm are much worse than the BTSE method, and the Feather algorithm is the worst one. Figure 4c shows that the WD-GDWE method preserves more spectral information than the other four algorithms. Figure 4d shows that it takes a little time to run the WD-GDWE, BTSE, Szeliski, and Feather algorithms, whereas the Dijkstra and Wavelet method are time-consuming. In a word, the WD-GDWE method is not only efficient, but also has a satisfactory effectiveness.

## 5. Conclusions

In this study, an efficient seam elimination method for UAV images based on Wallis dodging and Gaussian distance weight enhancement was proposed. The method has successfully tested by using UAV images acquired after the 5.12 Wenchuan Earthquake. By comparison with other five classical seam elimination methods, the conclusions from this study can be summarized as follows: (1) the WD-GDWE method can effectively adjust the brightness differences between two matched images; (2) the method can successfully eliminate the texture mosaic seams which are usually caused by geometric deformity, projection differences, and image capture position differences on UAV platforms; (3) the WD-GDWE method is highly-efficient, which can meet the high processing speed requirements of massive UAV images. Time-savings are very important in advancing the applications in the UAV industry, especially in emergency situations. The results of this study can be further extended to other fields, such as aerospace remote sensing and computer vision.

## Figures and Tables

**Table 1:** The parameters of the image sensor.

Items	Parameters
Image Sensor	Ricoh Digital
Pixel Number	3648 × 2736
Focal Distance	28 mm
CCD	1/1.75 inch
Navigation sensor	GPS
Image Format	JPEG

**Table 2:** Average of RMSE values of mean (M) and standard deviation (SD) calculated from the matched UVA images for stacking directly and Wallis dodging, respectively, in each type of land use.

Land Use		RMSE	
		M	SD
Building	Stacking Directly	24.5	6.5
	Wallis Dodging	0.0	0.2
Woodland	Stacking Directly	23.6	6.2
	Wallis Dodging	0.0	0.1
Farmland	Stacking Directly	19.8	5.7
	Wallis Dodging	0.0	0.1
Road	Stacking Directly	17.5	3.6
	Wallis Dodging	0.0	0.1
Water	Stacking Directly	36.2	9.5
	Wallis Dodging	0.0	0.3
